# Clinical Trial of the Outpatient Management of Pyelonephritis in Pregnancy

**DOI:** 10.1155/S1064744995000305

**Published:** 1995

**Authors:** Allyson M. Brooks, Thomas J. Garite

**Affiliations:** Department of Obstetrics and Gynecology University of California Irvine Medical Center 101The City Drive Orange CA 92668 USA

## Abstract

*Objective:* This study was designed to determine whether outpatient treatment of pyelonephritis in pregnancy can reduce costs without compromising safety or efficacy.

*Methods:* Pregnant patients with uncomplicated initial episodes of acute pyelonephritis were considered for outpatient management. The outpatient treatment consisted of an initial dose of IV ceftriaxone (2 g), followed by daily outpatient IM ceftriaxone (2 g) until resolution of fever and flank tenderness, followed by a 10-day course of oral antibiotics. The study group was compared with a group requiring inpatient treatment and a historical control group meeting the criteria for outpatient management but having been treated as inpatients in the previous year.

*Results:* Of the 34 treated as outpatients, only 4 (12%) required hospital admission and 1 developed an upper urinary tract recurrence. None of these patients had premature delivery or any other serious complication. The historical control group (N = 29) included 1 upper urinary tract recurrence, no preterm deliveries, and 1 case of acute respiratory disease syndrome. The outpatient group required an average of 3.4 daily outpatient visits compared with 3.9 days of hospitalization for the historical control group. The inpatient group (N = 39) was significantly more likely to require hospitalization >6 days (*P* = 0.0004), with a trend toward more frequent upper urinary tract recurrences (6/39 vs. 1/34, *P* = 0.08). The cost analysis revealed a 3-fold difference between outpatient and inpatient therapy ($1,100 vs. $3,350, *P* < 0.001).

*Conclusions:* The outpatient treatment of selected patients with pyelonephritis in pregnancy as a promising approach to reducing costs warrants further investigation.


Infectious Diseases in Obstetrics and Gynecology 3:50–55 (1995)
© 1995 Wiley-Liss, Inc.

### Clinical Trial of the Outpatient Management of Pyelonephritis in Pregnancy

**Allyson M. Brooks and Thomas J. Garite**

*Department of Obstetrics and Gynecology, University of California Irvine Medical Center, Orange, CA*

Abstract

***Objective:*** This study was designed to determine whether outpatient treatment of pyelonephritis in pregnancy can reduce costs without compromising safety or efficacy.

***Methods:*** Pregnant patients with uncomplicated initial episodes of acute pyelonephritis were considered for outpatient management. The outpatient treatment consisted of an initial dose of IV ceftriaxone (2 g), followed by daily outpatient IM ceftriaxone (2 g) until resolution of fever and flank tenderness, followed by a 10-day course of oral antibiotics. The study group was compared with a group requiring inpatient treatment and a historical control group meeting the criteria for outpatient management but having been treated as inpatients in the previous year.

***Results:*** Of the 34 treated as outpatients, only 4 (12%) required hospital admission and 1 developed an upper urinary tract recurrence. None of these patients had premature delivery or any other serious complication. The historical control group (N = 29) included 1 upper urinary tract recurrence, no preterm deliveries, and 1 case of acute respiratory disease syndrome. The outpatient group required an average of 3.4 daily outpatient visits compared with 3.9 days of hospitalization for the historical control group. The inpatient group (N = 39) was significantly more likely to require hospitalization >6 days ($P = 0.0004$), with a trend toward more frequent upper urinary tract recurrences (6/39 vs. 1/34, $P = 0.08$). The cost analysis revealed a 3-fold difference between outpatient and inpatient therapy ($1,100 vs. $3,350, $P < 0.001$).

***Conclusions:*** The outpatient treatment of selected patients with pyelonephritis in pregnancy as a promising approach to reducing costs warrants further investigation. © 1995 Wiley-Liss, Inc.

Key words

Preterm labor, preterm delivery, upper urinary tract infection, antibiotic therapy

Acute pyelonephritis complicates 1–2% of all pregnancies. It is the most common nonobstetric indication for antepartum hospitalization. Such patients are generally treated with IV antibiotics and kept hospitalized not only until their symptoms have resolved but 24–48 h beyond complete defervescence. The average hospital stay for this diagnosis is 3–5 days.[1] The risk of complications of pyelonephritis in pregnancy, especially preterm labor and delivery, as well as the need to prevent recurrences and permanent renal damage, have dictated this aggressive approach.[1] The recent emphasis on cost containment of health care and the shift toward outpatient management have caused clinicians to question the necessity of inpatient treatment of such common problems. Therefore, we designed this prospective study to determine whether the outpatient treatment of pyelonephritis in pregnancy can significantly reduce costs without compromising safety and efficacy.

### SUBJECTS AND METHODS

All pregnant patients with a diagnosis of acute pyelonephritis at the University of California Irv-

Address correspondence/reprint requests to Dr. Thomas J. Garite, Department of Obstetrics and Gynecology, University of California Irvine Medical Center, 101 The City Drive, Orange, CA 92668.

Presented at the Annual Clinical Meeting of the American College of Obstetricians and Gynecologists, Orlando, FL, 1994.

Received September 20, 1994

Accepted May 26, 1995

ine Medical Center were considered candidates for the study. The criteria for the diagnosis of pyelonephritis included a temperature ≥38°C, costovertebral angle tenderness (CVAT), and any one of the following: WBC >11,800 or suggestive symptoms, such as dysuria, flank pain, or nausea and vomiting.

Patients were excluded from outpatient management for any of the following reasons: evidence of sepsis (hypotension, obtundation, pulmonary edema, disseminated intravascular coagulation), temperature >39°C, severe nausea and vomiting, uncertain diagnosis, presence of immunocompromise [diabetes mellitus, human immunodeficiency virus (HIV), IV-drug abuse, or corticosteroid use], recurrent upper urinary tract infection, concomitant preterm labor, multiple gestation, severe penicillin allergy (not including rash only), history of renal disease or urinary tract anomalies, or presence of an indwelling bladder catheter.

Each patient eligible for outpatient treatment was observed for an initial 2-h period with a baseline laboratory evaluation (CBC, urinalysis with culture and sensitivity, and serum BUN and creatinine) and IV hydration with 1 l of a balanced crystalloid solution. This study was approved by the Medical Center's Institutional Review Board, and all patients were required to sign informed consents prior to their inclusion in the study. Once the diagnosis was confirmed and exclusion criteria were eliminated, 2 g of IV ceftriaxone (Rocephin®, Roche Laboratories, Nutley, NJ) was administered. This third-generation cephalosporin was chosen for its long half-life requiring administration only once every 24 h, its broad-spectrum coverage against common urinary pathogens, and its documented efficacy in the outpatient treatment of pyelonephritis and other serious infections in nonpregnant patients.[2–6] The patient was then seen the following day in the obstetrical clinic at which time 2 g of IM ceftriaxone was administered daily until the resolution of her fever and CVAT, followed by a 10-day course of oral antibiotics based on the culture and sensitivity results. Hospitalization was required for patients who were considered outpatient failures based on persistent fever or CVAT beyond 48 h of parenteral therapy, as well as for patients who developed nausea and vomiting, were noncompliant with clinic visits for parenteral antibiotics, showed evidence of preterm labor, or experienced adverse drug reactions. However, the requirements for delayed hospital admission in this group were not absolute; if the patient was clearly clinically improved at 48 h, she was continued on outpatient treatment.

Any patient excluded from outpatient management because of the above criteria (referred to as the inpatient group) received the traditional inpatient therapy consisting of hospitalization and the administration of parenteral antibiotics until she became afebrile and her CVAT resolved. This inpatient treatment was followed by a 10-day course of oral antibiotics based on urine culture and sensitivities. The admission laboratory evaluation included a CBC, catheterized urinalysis with culture and sensitivities, and serum BUN and creatinine. Blood cultures were done only on patients with suspected sepsis. A second antibiotic agent was added if the patient's fever persisted beyond 48 h of parenteral therapy.

The patients were followed prospectively for treatment failure, other serious complications [adult respiratory disease syndrome (ARDS), perinephric abscess, sepsis, or renal insufficiency], upper urinary tract recurrence, or premature delivery. Both groups (outpatient and inpatient) were compared with a historical control group which fulfilled the same criteria for outpatient management, yet had received traditional inpatient treatment in the year prior to the study period.

For the purpose of comparing the potential cost saving of outpatient management, only the hospital and outpatient costs were calculated. Included in the total costs for nonhospitalized patients were laboratory fees, emergency room visits for the initial evaluation, fetal assessment (NST, ultrasound), IV fluids, follow-up clinic visits, and pharmacy costs. The costs for hospitalized patients included hospital room fees, laboratory costs, emergency room and clinic visits, pharmacy costs, costs of supplies, and radiology fees. The costs of hospitalization for outpatent treatment failures were included in the analysis.

A statistical analysis was performed using the Mann-Whitney U-test, chi-squared, Fisher's exact test, and Student's t-test where appropriate. The significance was established using $P < 0.05$. The sample size was determined based on an 80% chance of showing a 50% reduction in cost with 95% confidence These calculations determined the need for

**TABLE 1. Patient demographics**

| | Outpatients (N = 34) | Historical controls (N = 29) | Inpatients (N = 39) |
|---|---|---|---|
| Age (years) | 23.2 ± 4.9 | 23.4 ± 5.9 | 23 ± 4.9 |
| Gravidity | 3.0 ± 2.1 | 2.4 ± 2.0 | 2.3 ± 1.5 |
| Parity | 1.4 ± 1.7 | 1.2 ± 1.9 | 0.9 ± 1.4 |
| EGA[a] | 20.1 ± 7 | 23.1 ± 7.1 | 26.1 ± 7.7 |
| Race (% Hispanic) | 85% | 93% | 77% |
| Prenatal care (% yes) | 35% | 38% | 62% |
| Insurance (% MediCal) | 50% | 41% | 72% |

[a]EGA = estimated gestational age.

**TABLE 2. Clinical characteristics of the outpatient and historical control groups**

| | Outpatients (N = 34) | Historical controls (N = 29) | *P* |
|---|---|---|---|
| Initial temperature (°C) | 37.8 ± 0.8 | 37.9 ± 0.8 | N.S.[a] |
| Pulse | 102.1 ± 16.3 | 100.4 ± 21.4 | N.S. |
| WBC | 13.6 ± 4.2 | 12.3 ± 3.7 | N.S. |
| Side (% right) | 91% | 66% | 0.01 |
| History of UTI[b] (% yes) | 26% | 28% | 0.92 |
| Organism (% *E. coli*) | 94% | 69% | 0.009 |

[a]N.S. = not significant.
[b]UTI = Urinary tract infection.

15 patients in each group. The power to demonstrate that no sacrifice in safety occurred with outpatient management was calculated retrospectively after the completion of study.

### RESULTS

From March 1, 1992, to March 31, 1993, 73 pregnant patients were diagnosed with acute pyelonephritis. Thirty-four (47%) met the criteria for outpatient management. Thirty-nine patients (53%) who were excluded from outpatient management composed the inpatient group. The reasons for exclusion included fever >39°C (15), significant nausea and vomiting (8), diabetes mellitus (5), recurrent upper urinary tract infection (5), uncertain diagnosis (2), and other (4). Twenty-nine controls (historical control group) were identified from a computerized data base as described in Subjects and Methods. All patients in the outpatient group received a full course of parenteral antibiotics, although some patients required phone calls to remind them to return for therapy. No patients were lost to follow-up until after delivery.

Table 1 is a demographic comparison of the 3 groups. There was no significant difference in parity, maternal age, gestational age at diagnosis, race, or prior prenatal care. The inpatient group was significantly more likely than the outpatient group to have government funding (MediCal) (72% vs. 41%, $P < 0.01$). The majority of patients in all groups were Hispanic; furthermore, the majority had received no prior prenatal care.

Table 2 is a comparison of the clinical characteristics between the outpatient and historical control groups. There was no difference in initial temperature, pulse, WBC, or prior urinary tract infection. The outpatient group was significantly more likely to have right-sided pyelonephritis (91% vs. 66%, $P = 0.01$) and to have *Escherichia coli* as the causative organism (94% vs. 69%, $P = 0.009$). No cephalosporin-resistant organism was isolated in either group.

**TABLE 3. Clinical characteristics of the outpatient and inpatient groups**

| | Outpatients (N = 34) | Inpatients (N = 29) | *P* |
|---|---|---|---|
| Pulse | 102.1 ± 16.3 | 105.2 ± 17.8 | N.S.[a] |
| WBC | 13.6 ± 4.2 | 14 ± 3.9 | N.S. |
| Side (% right) | 91% | 87% | 0.6 |
| History of UTI[b] (% yes) | 26% | 49% | 0.05 |
| Organism (% *E. coli*) | 94% | 56% | 0.0003 |

[a]N.S. = not significant.
[b]UTI = Urinary tract infection.

Table 3 is a comparison of the clinical characteristics between the outpatient and inpatient groups. There was no significant difference in the initial WBC or pyelonephritis related to a particular side. The inpatient group had a higher incidence of prior urinary tract infection (49% vs. 26%, $P = 0.05$) and the isolation of a wider variety of urinary pathogens (56% *E. coli* vs. 94% *E. coli*, $P = 0.0003$).

Table 4 depicts the urinary culture isolates of the 3 groups. A comparison of the duration of parenteral therapy needed to effect a resolution of the symptoms and signs of pyelonephritis showed that the outpatient group required an average of 3.4 ± 0.6 outpatient visits for evaluation or days of parenteral antibiotic therapy compared with 3.9 ± 2.4 days of hospitalization for the historical control group and 4.2 ± 1.3 days of hospitalization for the inpatient group.

**TABLE 4. Urinary isolates**

| | Outpatients N (%) | Historical controls N (%) | Inpatients N (%) |
|---|---|---|---|
| *E. coli* | 32 (94%) | 20 (69%) | 22 (56%) |
| *Klebsiella pneumoniae* | | 2 (7%) | 8 (20%) |
| *K. enterobacteria* | | | 1 (3%) |
| Staphylococcus | | 1 (3%) | 2 (5%) |
| Enterococcus | | | 2 (5%) |
| Enterobacteria | | 2 (7%) | 1 (3%) |
| Multiple organisms | | 3 (10%) | |
| Negative culture | 2 (6%) | 1 (3%) | 3 (8%) |

Of the 34 in the outpatient group, 4 required hospital admission for nausea and vomiting or persistent fever (12% treatment failure rate). None of these patients required prolonged hospitalization to effect a resolution of the acute infection (longest hospital stay was 4 days). One patient in the outpatient group developed a subsequent upper urinary tract infection with a different organism (*Enterobacter cloacae*) from the original infection (*E. coli*). No patients in the outpatient group had serious complications, other than recurrence. No perterm deliveries occurred and no cephalosporin-resistant organisms were isolated in this group. No patients were lost to follow-up.

Of the 29 historical controls, 4 (14%) required hospitalization >6 days. One patient developed an upper urinary tract recurrence. One patient developed ARDS on the second hospital day. This complication was felt to be iatrogenic because of volume overload. The patient responded to fluid restriction and supplemental oxygen, and assisted ventilation was not required. No preterm deliveries occurred, and no cephalosporin-resistant organisms were isolated.

Of the 39 patients excluded from outpatient management in the inpatient group, 8 (21%) required prolonged hospitalization >6 days to effect a cure. Six (15%) experienced an upper urinary tract recurrence. Two patients (6%) had other serious complications (1 had a perinephric abscess, and the other had transient renal insufficiency). Three patients (7%) had cephalosporin-resistant organisms (2 enterococcal infections and 1 methicillin-resistant staphylococcal infection). No preterm deliveries occurred in the inpatient group. Compared with the outpatient group, these hospital patients were significantly more likely to require prolonged therapy >6 days ($P = 0.0004$), with a trend toward more frequent upper urinary tract recurrences ($P = 0.08$).

A cost analysis demonstrated a 3-fold difference between outpatient and inpatient therapy ($1,100 vs. $3,350, $P < 0.001$). When all therapy for the episode of pyelonephritis was included, the outpatient group cost was $1,100 ± $1,105; the historical control group cost was $3,350 ± $2,150; and the inpatient group cost was $3,500 ± $1,235. The hospital charges (as opposed to costs) were not calculated, but the difference in the groups would be even more dramatic if these charges had been compared.

A retrospective analysis of the power to determine the safety of outpatient therapy showed that, with the sample size in this study, we achieved a 65% chance of showing a 2-fold increase in any serious complication with 95% confidence, and no increases in such complications were seen.

### DISCUSSION

Physicians and hospitals are being subjected to increasing pressures to reduce the cost of health care. One of the prime determinants of overall cost, especially of acute problems, is the need for and duration of hospitalization. In the past, only optimal health care, without much regard for cost, determined the need for hospitalization. In the case of pyelonephritis in pregnancy, with an appreciation of the major associated complications, particularly preterm labor and delivery, inpatient therapy with IV antibiotics and aggressive hydration became the standard of care. Recently, reports have appeared suggesting that patients with other serious acute infections, such as streptococcal endocarditis, childhood meningitis, and pyelonephritis in nonpregnant patients, can be safely and effectively treated as outpatients, especially with the availability of long-acting broad-spectrum antibiotics.[2–6] No previous reports have tested the possibility of similarly treated pyelonephritis in pregnancy although a recent study reported efficacy with oral antibiotics used in the inpatient setting.[7]

This first such study, although limited by the relatively small sample sizes, demonstrates that outpatient management of pyelonephritis in pregnancy substantially reduces the cost of treating this relatively common problem. Nearly 90% of the patients who met the inclusion criteria were successfully treated as outpatients with no serious complications, no preterm deliveries, and only 1 patient (3%) developing a recurrent urinary tract infection. These results were as good as or better than those seen with a reasonably similar historical control group previously treated as inpatients, who otherwise would have been eligible by these criteria for outpatient management.

With our conservative protocol, we were still able to treat nearly half of all patients presenting with pyelonephritis at an average cost savings of more than $2,200/patient. The savings would be even greater in the private setting in which many of these patients would have their initial evaluation and treatment in the physician's office.

One might question the criteria we chose to define pyelonephritis, specifically allowing the inclusion of patients with negative cultures. Since cultures are not available at the time the diagnosis must be made and few clinicians use urine Gram stains, we felt that it would not be appropriate to eliminate these patients, since they had prospectively consented and had been included in the study protocol. Similarly, we allowed the inclusion of all patients with identical criteria in the 2 control groups. However, eliminating the few patients with negative cultures would not have changed the results or conclusions of this study.

The primary limitation of this study is our inability to make more definitive statements about the safety of outpatient therapy because of the relatively small sample size studied. Therefore, this study must be viewed as a preliminary report suggesting promising results and requiring corroboration by subsequent larger randomized studies to establish outpatient therapy as a safe alternative. The purpose of a retrospective power analysis is not to give scientific credibility to the sample size but to give the reader an impression of the magnitude of difference in complications that a study of this sample size might be able to demonstrate. Although large differences in the rates of any serious complications expected to be seen in this study were not, larger studies are needed to determine if any compromise in patient safety results from such a treatment protocol. It is important to emphasize that hospitalization itself is not necessarily the safest alternative. In a previous report, we discovered a surprisingly high rate (8%) of pulmonary edema and ARDS in hospitalized patients with acute pyelonephritis in pregnancy.[8] A multifactorial analysis determined that excess IV fluid administration and parenteral tocolytic use iatrogenically contributed to this serious complication. A similar complication seen in this study occurred in a patient from the historical control group who appeared to have received excessive hydration. Nonetheless, it is important that we scrutinize the safety and efficacy of such outpatient protocols in a carefully controlled setting before these protocols are imposed on us by third-party payers.

Because of this concern, we used relatively stringent and conservative exclusion criteria. These exclusion criteria, especially fever >39°C and severe nausea and vomiting, were the reason that the outpatient treatment could be applied to fewer than half of the patients presenting with this diagnosis. Home health nursing, including outpatient IV therapy, may make it possible to extend such treatment to the excluded patients as well. However, the results of this study indicate that the exclusions were appropriately chosen, as both the serious complications and the vast majority of prolonged hospitalizations and recurrences occurred in the inpatient group, which met the exclusion criteria.

The availability of newer, long-acting, safe antibiotics with a broad-spectrum of activity against pathogens found in urinary tract infections makes this type of study feasible. Ceftriaxone is such an antibiotic. It is a broad-sprectrum, third-generation cephalosporin that when administered IM is completely absorbed, reaching peak plasma concentrations in 2–3 h, and eliminated in a half-life of 6–9 h. Two-thirds of this drug is excreted in the urine, and urinary concentrations of 40% of the peak concentrations are maintained for 24 h. It has an excellent spectrum of activity against commonly occurring gram-negative and gram-positive urinary pathogens.[9] Although the experience of cephalosporins in pregnancy has generally demonstrated safety, this relatively new cephalosporin is listed as a "Pregnancy Category B" with no evidence of harm in high-dose animal studies and limited experience in human pregnancies.[10] Safety in neonates, however, has been established.

Optimally, all cases of pyelonephtitis should be prevented by early prenatal care with screening and treatment of asymptomatic bacteriuria.[1] As seen in this study, the vast majority of our patients presented with pyelonephritis prior to their first prenatal visit. Surprisingly, we were able to convince all patients to follow the protocol and keep their appointments once the diagnosis had been made. Nonetheless, many patients do not seek prenatal care early enough, if at all. Even under good prenatal care, patients all too often develop acute upper urinary tract infections. Even screening all patients at the first prenatal care visit will not eliminate all such cases. Gilstrap et al.[12] have shown that as many as one-third of all significant bacteriurias will occur later in pregnancy in women with initially negative cultures and about 25% or more of these will develop pyelonephritis if the condition goes undetected and untreated. Therefore, selective cost-savings protocols are needed to deal with this common antepartum diagnosis that adds substantially to the cost of maternity and newborn care.

This preliminary study does demonstrate that a selective approach to outpatient management of acute pyelonephritis in pregnancy can substantially reduce the cost of treating this entity without apparently compromising patient safety. Larger studies are needed to document the safety of such an outpatient management scheme before such an approach is accepted and applied.

### REFERENCES

1. Martens MG: Pyelonephritis. Obstet Gynecol Clin North Am 16(2):305–315, 1988.
2. Francioli E, Etienne J, Hoigne R, Thys JP, Gerber A: Treatment of streptococcal endocarditis with a single daily dose of ceftriaxone sodium for four weeks. JAMA 267: 264–276, 1991.
3. Baumgartner JD, Glauser MP: Single daily dose treatment of severe refractory infections with ceftriaxone: Cost savings and possible parenteral outpatient treatment. Arch Intern Med 143:1868–1873, 1994.
4. Russo TA, Cook S: Intramuscular ceftriaxone in home parenteral therapy. Antimicrob Agents Chemother 32: 1439–1440, 1988.
5. Bradley JS, Ching DK: Outpatient therapy of serious pediatric infections with ceftriaxone. Pediatr Infect Dis J 7:160–164, 1980.
6. Karachalios G, Georgiopoulos AN, Kinztziou H: Treatment of acute pyelonephritis in women with intramuscular ceftriaxone: An outpatient study. Chemotherapy 37: 292–296, 1991.
7. Angel J: Acute pyelonephritis in pregnancy. A prospective study of oral versus intravenous antibiotic therapy. Obstet Gynecol 76:28, 1990.
8. Towers CV, Kaminskas CM, Garite TJ, et al: Pulmonary injury associated with antepartum pyelonephritis. Can patients be identified? Am J Obstet Gynecol 163:773–775, 1991.
9. Scully BE, Fu KP, Nev HC: Pharmacokinetics of ceftriaxone after intravenous and intramuscular injection. Am J Med 77(Suppl):112–115, 1984.
10. Roche Laboratories: Product Insert: Rocephin. Nutley, NJ: Roche Laboratories, 1994.
11. James J, Mulnall A, de Louvois J: Ceftriaxone: Clinical experience in the treatment of neonates. J Infect 2:25–33, 1985.
12. Gilstrap LC, Cunningham FG, Whaller PJ: Acute pyelonephritis in pregnancy: An anterospective study. Obstet Gynecol 57:409–413, 1981.
